# Eccentric distance zone analysis system: New regional evaluation of cephalic fixator tip location for predicting cut-out in geriatric intertrochanteric fractures with internal fixation

**DOI:** 10.3389/fsurg.2022.956877

**Published:** 2022-10-18

**Authors:** Yun-fa Yang, Jian-wen Huang, Xiao-sheng Gao, Zhong-he Xu

**Affiliations:** Department of Orthopaedic Surgery, Guangzhou First People's Hospital, the Second Affiliated Hospital, School of Medicine, South China University of Technology, Guangzhou, China

**Keywords:** eccentric distance zone analysis system, intertrochanteric fractures, internal fixation, cut-out, artificial intelligence

## Abstract

**Objective:**

The aim of this study was to investigate an eccentric distance (ED) zone analysis system for regional evaluation of the cephalic fixator tip based on the ED of the cephalic fixator tip referenced to the radius of its own femoral head to predict cut-out in intertrochanteric fractures (ITF) with internal fixation.

**Methods:**

First, we assumed all the femoral heads were regular spheres with the radius (*R*_FD_) of “3” for a complete match of the Cleveland zone system and calculated the ED of the cephalic fixator tip by measuring the distances from the cephalic fixator tip to the geometric central axis in the femoral neck and head on both anteroposterior (AP) view and lateral view radiographs. Second, we defined the maximum transverse section of the femoral head into three zones named ED Zone A with ED less than “1,” Zone B with ED ranging in “1–2,” and Zone C with ED ranging in “2–3” in turns by concentric circles (circles A, B, and C) with the radius of 1/3, 2/3, and 3/3 times of *R*_FD_, respectively. Third, we evaluated the ED zones according to the ED and location of the cephalic fixator tip in the eligible 123 ITF patients with single-screw cephalomedullary nail (SCMN) fixation and then analyzed the correlation between the cut-out rate and the ED zones.

**Results:**

The cut-out rates in ED Zones A, B, and C were 4.17%, 38.46%, and 100%, respectively. Multivariate logistic regression indicated that ED Zone A had at least a 14 times lower rate of cut-out compared with ED Zone B. The cephalic fixator tip located in ED Zone A has a lower cut-out rate than that in Cleveland Zone 5. The cut-out rate in ED Zone A is significantly lower than that in the region inside Cleveland Zone 5 but outside ED Zone A.

**Conclusion:**

ED zone analysis system is a reliable regional evaluation of the cephalic fixator tip position for predicting cut-out in geriatric ITF patients with SCMN fixations and potentially an artificial intelligence measurement during surgery. For decreasing the cut-out rate, the cephalic fixator tip should be located in ED Zone A.

## Introduction

The incidence of intertrochanteric fractures (ITF) is increasing every year due to the aging population globally. The social burden of ITF significantly increases because of their existing comorbidities, mortality, and bedridden complications resulting in aging (1, 2). Generally, surgical treatment is the first choice for geriatric ITF patients unless there are contraindications to surgery. Nowadays, cephalomedullary nails have been commonly used for ITF due to their biomechanical advantages and good clinical outcomes. However, postoperative implant failures (such as cut-out), which occur in 1.85%–20.5% (1, 3–6), remain a great challenge to orthopedists.

Actually, cut-out is highly associated with the placement of the cephalic fixator tip (7–15). Furthermore, the cephalic fixator should locate in the geometric center of the transversal surface of the femoral head. Kyle et al. suggested that a cephalic fixator should place centrally within the femoral head because the region was the connection area of compression and tension trabeculae (16). Jenkins et al. demonstrated that the strongest and thickest trabecular bone was in the center of the femoral head by microarchitectural evaluation (17), and optimal fixation would be achieved if the cephalic fixator was placed at the neck axis and the center of the femoral head (17). Similarly, Liu et al. confirmed that the highest bone mineral density (BMD) of the proximal femur was in the femoral head, particularly in the middle of the femoral head by quantitative computed tomography, which showed that the cephalic fixator should be placed in the central region of the femoral head for maximum holding power (18). Actually, the Cleveland zone system is easily available for the surgeon to evaluate the intraoperative cephalic fixator placement (3, 16, 17, 19–22).

However, cut-out still occurs in patients who had the cephalic fixator tip in Cleveland Zone 5. Our previous study has confirmed that the probability of cut-out increased dramatically with the increase of eccentric distance (ED) of the cephalic fixator tip, and the best cut-off value of ED for predicting cut-out is “1.022” with a sensitivity of 73.3% and a specificity of 86.1% by the receiver operating characteristic (ROC) analysis (area under the curve, AUC = 0.867, *p* < 0.001) when we assumed all the femoral heads were regular spheres and the radius (*R*_FD_) as “3” for a complete match of the Cleveland zone system (23). The mechanical effect of the cephalic fixator tip in Cleveland Zone 5 is different because the ED of the marginal region of Cleveland Zone 5 is much bigger, and that is probably why cut-out still occurs in patients for whom the cephalic fixator tip located in Cleveland Zone 5. Consequently, the geometric center region in the femoral head should be a circle but not a square. Because on a central–central principle in an approximate sphere—the femoral head, a circling zone is better to describe the center zone than a square zone of the Cleveland zone system. We need the right tool to evaluate the cephalic fixator tip position to ensure that the cephalic fixator tip is exactly located in the geometric center region in the femoral head to prevent cut-out.

Therefore, we hypothesized that the placement of cephalic fixator tips with different EDs should have different cut-out risks. We aimed to (1) design an ED zone analysis system for measurement of cephalic fixator tip position, (2) look for the optimal center–center region of the femoral head, and (3) potentially verify the artificial intelligence (AI) applicability of the ED zone analysis system in predicting the cut-out rate in ITF patients with internal fixation during surgery.

## Materials and methods

We designed an ED zone analysis system and analyzed the correlation between the cut-out rate and the ED zones of the cephalic fixator tip location in ITF patients with internal fixation.

### ED zone analysis system

First, we assumed the femoral head was a regular sphere and standardized all the radius of the femoral head (*R*_FD_) to be “3” (*R*_FD_ = “3”) no matter how big the *R*_FD_ was for a complete match of the Cleveland zone system, easy comparison of the ED, and convenient identification for artificial intelligence based on the study by Yang et al. (23).

**Figure 1:**
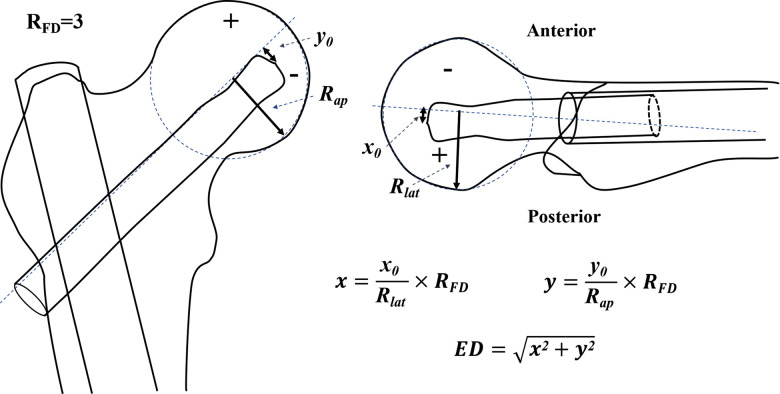
The measurement and calculation of ED based on our previous study (23). No matter how big the radius of the femoral head (*R*_FD_) was, we standardized the *R*_FD_ as “3” (*R*_FD_ = “3,” without any unit) for a good match with the Cleveland zone system and easy calculation. “*x*_0_*, y*_0_, *R_ap_*, *R*_lat_” were the actual measured values. The ED of the cephalic fixators was calculated by the distances from the cephalic fixator tip to the geometric central axis in the femoral neck and head on both the AP view radiograph (*y = y*_0_*/R_ap_* × *R*_FD_) and the lateral view radiograph (*x = x*_0_*/R_lat_* × *R*_FD_) resulted in ED = (*x*^2 ^+ *y*^2^)^1/2^. The value of “*x*” or “*y*” was defined as positive if the cephalic fixator tip was on the superior or posterior, and as negative if the cephalic fixator tip was on the inferior or anterior when compared with the axis of femoral head. ED, eccentric distance.

**Figure 2:**
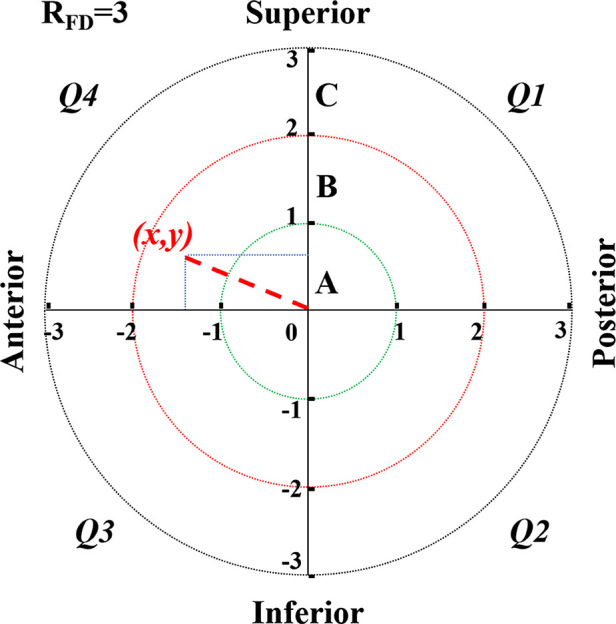
ED zone analysis system is just like the coordinate graph (an *x–y* plot). In this system, no matter how big the radius of the femoral head (*R*_FD_) was, we standardized the *R*_FD_ as “3” (*R*_FD_ = “3,” without any unit) for a good match with the Cleveland zone system and easy calculation. We defined the femoral head into three zones named Zone A (remote zone), B (subcenter zone), and C (center zone) in turns by three concentric circles (circle A, B, and C) with the radius of 3/3, 2/3, and 1/3 times of *R*_FD_, respectively (*R*_A_, *R*_B,_ and *R*_C_ was “3,” “2,” and “1,” accordingly). The femoral head was divided into four quadrants (Q1, 2, 3, and 4). ED of the tip point (*x,y*) is the distance from the circle center to the point of (*x,y*). ED, eccentric distance.

Second, we calculated the ED of the cephalic fixator tip using the distances from the cephalic fixator tip to the geometric central axis in the femoral neck and head on both lateral view radiograph (*x = x*_0_*/R*_lat_ × *R*_FD_) and anteroposterior (AP) view radiograph (*y = y*_0_*/R_ap_* × *R*_FD_) that resulted in “ED = (*x*^2^+*y*^2^)^1/2^” based on our previous study (Figure 1) (23). The femoral neck geometric central axis was a straight line through both the femoral head geometric center and the femoral neck geometric center (24). “*x*_0_, *y*_0_, *R_ap_*, *R*_lat_” were actual measured values. The value of “*x*” or “*y*” was defined as positive if the cephalic fixator tip was on the superior or posterior, and as negative if the cephalic fixator tip was inferior or anterior referencing the axis of the femoral head. Thus, we could intuitively locate the tip of the cephalic fixator in the coordinate diagram of the femoral head and easily calculate the ED. ED of the tip point (*x,y*) is the distance from the circle center to the point of (*x,y*). (Figure 2)

Third, we designed the ED zone analysis system. In this system, we defined the maximum transversal section of the femoral head into three zones named ED Zone A (center zone), B (subcenter zone), and C (remote zone) in turns by three concentric circles (circles A, B, and C) with the radius of 1/3, 2/3, and 3/3 times of *R*_FD_, respectively (*R*_A_, *R*_B,_ and *R*_C_ were “1,” “2,” and “3” accordingly because *R*_FD_ = “3”) for evaluating the location of cephalic fixator tip. Therefore, the ED in Zones A, B, and C ranged as “0–1,” “1–2,” and “2–3,” respectively. To accurately analyze the cephalic fixator tip placements, we could further subdivide the femoral head into four quadrants (Q1, 2, 3, and 4) in this system (Figure 2).

### Primary verification of ED zone analysis system in geriatric ITF patients

We verified the ED zone analysis system in patients with ITF treated surgically and followed up in our hospital between September 2016 and August 2020 (approved by the Ethics Committee of our Hospital) retrospectively. There were 187 ITF patients who were treated and followed up in our hospital during this period.

The exclusion criteria are as follows: (1) age <65 years, (2) pathological fractures, (3) loss of preoperative or postoperative radiographs, (4) internal fixation was dual-screw cephalomedullary nail or plate system, and (5) patients without any implant failures during radiological follow-up of less than 6 months.

**Figure 3:**
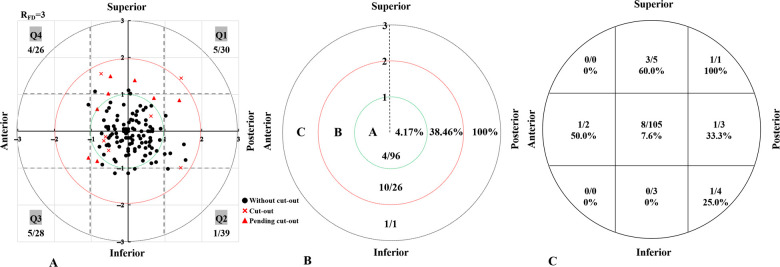
The distribution of cephalic fixator tip placements according to the ED zone analysis system (**A**), the rate of cut-out in each zones based on ED zone analysis system (**B**), and the rate of cut-out in each zones based on Cleveland zone system (**C**). Cephalic fixator position in ED Zone A has lower cut-out rate than that in the Cleveland Zone 5 (cut-out rate: *R*_EDZoneC _= 4.17%, *R*_ClevelandZone5 _= 7.62%). The cut-out rate in ED Zone A is significant lower than that in the region inside the Cleveland Zone 5 but outside the ED Zone A (*p *= 0.0016). ED, eccentric distance.

The eligible ITF patients were divided into the Cut-out group and the Non-Cut-out group according to whether the cephalic fixator cut-out or not. We located the tip of the cephalic fixator in the coordinate diagram (an *x–y* plot) of the femoral head and evaluated the cephalic fixator tip position by individually measuring the ED of the cephalic fixator tip in ITF patients with single-screw cephalomedullary nail (SCMN) fixation. Then, we assessed all the cephalic fixator tip positions in ED Zones A, B, and C according to the ED of the cephalic fixator tip. Finally, we analyzed the correlation between the cut-out rates and the ED zones (Figure 3).

The clinical data including age, gender, fracture site, fractures classifications according to the AO Foundation and Orthopaedic Trauma Association system (AO/OTA), American Society of Anesthesiologists (ASA) classification, anesthesia, fixation type, reduction quality, Cleveland zone system, and ED zones of cephalic fixator tip (cephalic fixator tip position based on ED zone analysis system) were analyzed.

All the radiological parameters were evaluated by two observers (J-wH and X-sG). Fracture classification was determined using preoperative AP radiographs by the AO/OTA system (2018 version) (25). Bone qualities were evaluated using the Singh index on preoperative AP radiographs (26). Reduction qualities were graded into three conditions (poor, acceptable, and good) based on the criteria developed by Baumgaertner et al. (27).

The relation between the ED zones and the cut-out rate (the rate of cut-out and pending cut-out) was analyzed. The definition of cut-out was the upper extrusion of the cephalic fixator from the femoral head. The pending cut-out was the presence of over 20° decrease of neck-shaft angle (NSA) on the AP view with no cephalic fixator penetration in the last radiographic follow-up compared with the NSA at the first radiograph right after the surgery.

### Statistical analysis

The occurrence of cut-out was defined as the dependent variable. Univariate analysis of continuous and categorical variables was performed using Student’s *t*-test and *χ*^2^ test, respectively. All of the significant variables in the univariate analysis (*p* < 0.1) and potential variates (such as age, gender, fracture type, and reduction quality if *p* < 0.2 in univariate analysis) were entered into multivariate logistic models. The Hosmer–Lemeshow goodness-of-fit test (H–L test) was used to evaluate if the models fit the data. The fitting curve was used for the correlation between the ED zones and the probability of cut-out. All analyses above were performed using SPSS (IBM SPSS Statistic for Windows, Version 25.0, IBM Corp, Armonk, NY, United States). All tests were two-sided, and statistical significance was defined as the *p*-value below 0.05. The ROC curves were performed to assess the cut-off value and the reliability of the ED zone analysis system in predicting the cut-out rate with MedCalc® Statistical Software version 19.5.6 (MedCalc Software Ltd, Ostend, Belgium).

## Results

A total of 123 eligible geriatric ITF patients with SCMN fixation were included in this full analysis. The 123 patients (43 males and 80 females) aged 80.4 ± 8.4 years. The mean follow-up was 11.8 months (range, 6–48 months). Overall, 15 ITF patients were found with a cut-out (7 of cut-out and 8 of pending cut-out, Cut-out group). The remaining 108 ITF patients were without cut-out (Non-Cut-out group).

The cephalic fixator tip placement evaluated by the ED zone analysis system and Cleveland zone system are shown in Figure 3. In the ED zone analysis system, 96 cephalic fixator tips (in 96 hips of 96 patients) were located in ED Zone A (center zone), 26 cephalic fixator tips (in 26 hips) in ED Zone B (subcenter zone), and 1 cephalic fixator tip (in 1 hip) in ED Zone C (remote zone). The cut-out rates in ED Zones A, B, and C were 4.17%, 38.46%, and 100%, respectively. The cephalic fixator tip position in ED Zone A has a lower cut-out rate than that in Cleveland Zone 5 (cut-out rate: *R*_EDZoneA _= 4.17%, *R*_ClevelandZone5 _= 7.62%). The cut-out rate in ED Zone A is significantly lower than that in the region inside Cleveland Zone 5 but outside ED Zone A (Fisher exact test, *p *= 0.0016) (Figure 3).

In the univariate system (Table 1), no significant differences were found in age, gender, fracture site, fracture classification, anesthesia, ASA classification, fixation type, and reduction quality. Cephalic fixator placements evaluated by the ED zone analysis system had significant differences for cut-out (*p* < 0.001).

**Table 1:** Univariate analysis of collected data.

Factor	Overall (*n* = 123)	Non-Cut-out group (*n* = 108)	Cut-out group (*n* = 15)	*p*-value	OR (95% CI)
Age (mean ± SD)	80.4 ± 8.40	80.3 ± 8.43	81.1 ± 8.46	0.744^a^	1.01 (0.95–1.08)
Gender				0.255^b^	2.35 (0.63–8.84)
Male	43 (35.0)	40 (37.0)	3 (20.0)		
Female	80 (65.0)	68 (63.0)	12 (80.0)		
Fracture site				0.781^b^	1.27 (0.43–3.77)
Left	71 (57.7)	63 (58.3)	8 (53.3)		
Right	52 (42.3)	45 (41.7)	7 (46.7)		
AO/OTA classification				0.108^b^	NA
31A1	62 (50.4)	58 (53.7)	4 (26.7)		
31A2	56 (45.5)	46 (42.6)	10 (66.7)		
31A3	5 (4.1)	4 (3.7)	1 (6.6)		
Anesthesia				0.598^b^	1.96 (0.42–9.27)
Spinal	95 (77.2)	82 (75.9)	13 (86.7)		
General	28 (22.8)	26 (24.1)	2 (13.3)		
ASA				0.719^b^	NA
2	54 (43.9)	46 (42.6)	8 (53.3)		
3	66 (53.7)	59 (57.4)	7 (46.7)		
4	3 (2.4)	3 (2.8)	0 (0.0)		
Fixation type (%)				0.559^b^	1.39 (0.46–4.21)
Blade	41 (33.3)	35 (32.4)	6 (40.0)		
Screw	82 (66.7)	73 (67.6)	9 (60.0)		
Reduction quality				0.176^b^	NA
Good	54 (43.9)	50 (46.3)	4 (26.7)		
Acceptable	47 (38.2)	38 (35.2)	9 (60.0)		
Poor	22 (17.9)	20 (18.5)	2 (13.3)		
Cleveland zone system				**0.002** ^b^	6.39 (1.99–20.57)
Zone 5	105 (85.4)	97 (89.8)	8 (53.3)		
The other zones	18 (14.6)	11 (10.2)	7 (46.7)		
ED zone analysis system				**<0.001** ^b,c^	15.81 (4.48–55.83)
Zone A	96 (78.0)	92 (85.2)	4 (26.6)	**<0.001** ^b,d^	14.38 (4.02–51.55)
Zone B	26 (21.2)	16 (14.8)	10 (66.7)		
Zone C	1 (0.8)	0 (0)	1 (6.7)		

AO/OTA, AO Foundation and Orthopaedic Trauma Association; ASA, American Society of Anesthesiologists; ED, eccentric distance; OR, odds ratio; CI, confidence interval; NA, not applicable.

The bold values represent significant difference between the two groups.

^a^ Student’s t-test for continuous variables.

^b^ Chi-square test for categorical variables.

^c^ The reference category is Zone A (comparing with Zone B and C).

^d^ The reference category is Zone A (comparing with Zone B).

In the multivariate analysis, the age, gender, fracture type, reduction quality, and ED zone system were included. Only the ED zone analysis system was independently associated with the cut-out (Table 2). The ED Zone B (subcenter zone) had an over 14 times higher rate of cut-out when compared with the corresponding center zone [ED Zone A, adjusted odds ratio (OR) = 14.38, 95% confidence interval (CI), 4.02–51.55, *p* < 0.001].

**Table 2:** Multivariate logistic regression analysis.

Factor	*β* value	*p-*value	Adjusted OR	95% CI lower	95% CI upper
Unstable fracture	0.470	0.540	1.600	0.355	7.201
poor reduction	1.110	0.495	3.033	0.125	73.665
Cleveland noncentral zone^a^	1.874	0.119	6.513	0.617	68.790
ED noncentral zone^b^	2.305	**0.003**	10.026	2.236	44.950

ED, eccentric distance; OR, odds ratio; CI, confidence interval.

The bold values represent significant difference between the two groups.

^a^ Cleveland noncentral zone means the other zones except zone 5, the reference category is Cleveland zone 5.

^b^ ED noncentral zone means ED zone C and B, the reference category is ED zone A.

**Figure 4:**
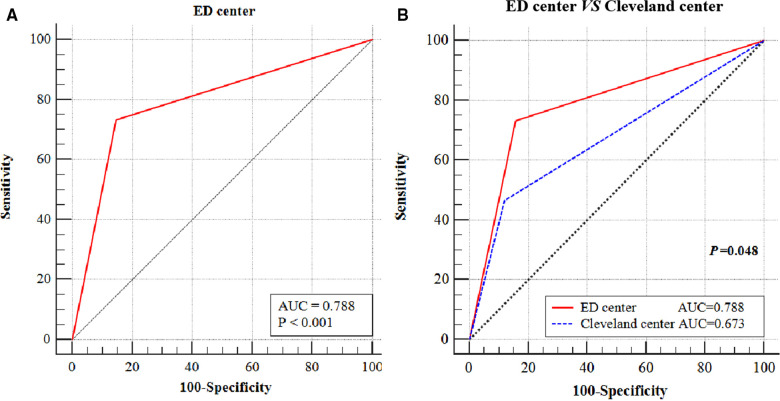
The graph shows the ROC analysis of ED zone C. The ED center (ED Zone C) was reliable in predicting cut-out (AUC = 0.788, *p *< 0.001) (**A**). Compared with the Cleveland center zone (Zone 5), ED center zone (ED Zone C) had significant low cut-out rate (AUC of ED center was 0.788; AUC of Cleveland center was 0.673; *p *= 0.048) (**B**). ROC, receiver operating characteristic; ED, eccentric distance; AUC, area under the curve.

The diagnostic effect of ED zone A, of which the AUC was 0.788 (*p* < 0.001), indicated that the cephalic fixator tip position located in ED Zone A (center zone) could significantly reduce the cut-out rate (Figure 4A). Compared with the Cleveland center zone (Zone 5), ED center zone (ED Zone A) had significant low cut-out rate (AUC of ED center was 0.788; AUC of Cleveland center was 0.673; *p *= 0.048) (Figure 4B). Compared with the Cleveland noncentral zone (all the zone outside of Zone 5), Cleveland center (Zone 5) has no significant low cut-out rate by multivariate logistic regression analysis (Adjusted OR = 6.513; 95% CI, 0.617 to 68.790; *p *= 0.119). However, the ED center (ED Zone A) has a significant low cut-out rate in comparing with the ED noncentral zone (Zone C and B) (Adjusted OR = 10.026; 95% CI, 2.236 to 44.950; *p *= 0.003) (Table 3).

**Table 3:** Reliability between two independent observers for measuring variables.

Variable	ICC or *κ*	95% CI	Reliability
Singh index	0.682	0.573–0.767	Excellent
Fracture classification	0.788	0.709–0.847	Excellent
Reduction quality	0.809	0.709–0.909	Almost perfect
Cleveland zone	0.669	0.565–0.773	Excellent
Cleveland center	0.745	0.641–0.829	Excellent
ED value	0.943	0.701–0.978	Almost perfect
ED center	0.763	0.624–0.902	Excellent

ED, Eccentric distance; CI, confidence interval; ICC, intraclass correlation coefficient; κ, Kappa coefficient.

We made further subdivisions in ED Zone B. In terms of the subdivided ED Zone B, the cut-out rates in the ED Zones B_1_, B_2_, B_3_, and B_4_ were 50% (3/6), 10% (1/10), 50% (3/6), and 66.7% (4/6), respectively.

## Discussion

The occurrence of the cut-out in geriatric ITF with cephalomedullary nailing is highly associated with implant placement, particularly the location of the cephalic fixator tip within the femoral head. The center–center principle was the leading principle of the cephalic fixator tip position (3, 17, 28, 29). However, precise tools were still lacking to measure the real center region in the femoral head in the literature. Thus, we design a new evaluation tool based on measuring the ED of the cephalic fixator tip, the ED zone analysis system, to resolve the problems above and verify its reliability. In this study, we find that the ED zone analysis system is a reliable evaluation tool for the measurement of the cephalic fixator tip position in predicting the cut-out rate in geriatric ITF patients with SCMN fixation. The rate of cut-out rises with the increasing ED. Clinically, the “real” center region should be in Zone A based on the ED zone analysis system. We can potentially use the ED zone analysis system in artificial intelligence measurements just during internal fixation surgeries.

### ED zone analysis system can precisely predict the cut-out rate

The cut-out rates in ED Zones A, B, and C were 4.17%, 38.46%, and 100%, respectively. ED center zone (ED Zone A) had at least a 14 times lower rate of cut-out compared with the ED subcenter zone (ED Zone B) by multivariate logistic regression (*p *< 0.001). Positioning the cephalic fixator tip in the femoral head as centrally as possible could decrease the cut-out rate even if it was accompanied by the slightly superior or anterior placement. Moreover, the “slightly superior or anterior” can be determined quantitatively by this system. Therefore, the ED zone analysis system is significantly accurate for predicting cut-out.

### ED zone A may be the best location of the cephalic fixator tip

Our previous study has confirmed that the probability of cut-out increased dramatically with the increase of ED, and the best cut-off value of ED for predicting cut-out is “1.022” with a sensitivity of 73.3% and a specificity of 86.1% by the ROC analysis (23). All the ED in ED Zone A (ED center) are less than “1” (less than the best cut-off value of ED), and the cut-out rate in ED Zone A was only 4.17% (in other words, the cephalic fixator tip placed in ED Zone A had a non-cut-out rate over 95%). The cephalic fixator tip located in ED Zone A had at least a 14 times lower rate of cut-out compared with that in the ED subcenter zone (ED Zone B) by multivariate logistic regression (*p *< 0.001). Consequently, ED Zone A may be the best location for the cephalic fixator tip.

Furthermore, ED Zone A showed higher reliability than the Cleveland Zone 5 dose. First, compared with the Cleveland center zone (Zone 5), ED Zone A had a significantly low cut-out rate by ROC analysis (AUC: 0.788 vs. 0.673; *p *= 0.048). There is some difference in biomechanics heterogeneity in ED Zone A when compared with Cleveland Zone 5 because ED Zone A covers just the region of the internally tangent circle of Cleveland Zone 5. In comparison with ED Zone A, the biomechanical effects of cephalic fixator tips in the nonoverlapping parts of the two zones, the margins of Cleveland Zone 5, are probably more similar to the adjacent regions of other noncentral zones. The cephalic fixator tips located in these margin regions have a much higher risk of secondary movement or rotation than those in ED Zone A. Second, based on the central–central principle, ED Zone A is more intuitive and easier to understand and fit with observation habits in describing the geometric center of the femoral head than Cleveland Zone 5. In this study, we found that four in nine cases (4/9, 44.44%) with a cut-out located in the region of Cleveland Zone 5 while just outside of ED Zone A.

Considering that different cephalic fixator tip placements had the same ED, we further subdivided ED Zone B into four quadrants for better clinical usage. We found the low rate of cut-out was in the inferior-posterior region (ED Zone B_2_). Many studies had also demonstrated that central or inferior on AP view and central or posterior on lateral view within the femoral head were optimal options to prevent cut-out (13, 27, 29–33). The reason for the discrepancies between the previous conclusions and our results probably is that not all the cases with cut-out previously were in ED Zone B_2_ but the more marginal locations with much bigger ED. As for the other regions of ED Zone B (ED Zones B_1_, B_3_, and B_4_), there were no significant differences in the cut-out rate, which may be attributed to the small number of cases (only 26 cases in ED Zone B).

In terms of the ED Zone C, it was excessively eccentric to place the cephalic fixator tip in this region. Only one case was found in ED Zone C, which was observed with cut-out. With the assistance of the C-arm and cephalic fixator insertion principle, an overlarge ED was almost impossible in clinical practice. We should not finish the operation with an extremely ED of cephalic fixator tip position on AP or lateral view unless the patient's poor general condition.

Therefore, the ED Zone A could be the excellent position of the cephalic fixator tip.

### ED zone analysis system may potentially be an AI application during surgery

The ED zone analysis system can be easily used because the measurement and the numerical relationship of the ED are completely matched the Cleveland zone system and the calculation of the ED only based on the AP view and the lateral view radiographs. In addition, the ED is a relative value measurement (the measurement with no complicated formula, regardless of magnification), which provides convenience in clinical usage. If we can set up the relative software of the ED zone analysis system in the C-arm x-ray machine, we may even use the ED zone analysis system in AI measurement just during surgeries.

### Limitations or weaknesses

However, there are still some limitations or weaknesses in this study. First, the design of ED zones is based on the ideal condition that the femoral head is a regular sphere. Second, we only verify the applicability of the ED zone analysis system in the single-screw cephalomedullary nails, resulting in the conclusion that may not be suitable for other types of internal fixations for the treatment of ITF. Third, we have not considered the quantitative osteoporosis assessment of the femoral head and the depth of cephalic fixator tips in the femoral head in geriatric ITF patients accurately. Thus, further studies are necessary to verify the clinical applicability of the ED zone analysis system and its clinical significance.

## Conclusions

The ED zone analysis system is a new reliable evaluation tool and potentially an AI application for measuring the cephalic fixator tip position in predicting cut-out in geriatric ITF patients with SCMN fixation. The cut-out rate rises with increasing ED. For decreasing the cut-out rate, the cephalic fixator tip should be located in ED Zone A (the center of the femoral head).

## Data Availability

The raw data supporting the conclusions of this article will be made available by the authors, without undue reservation.
